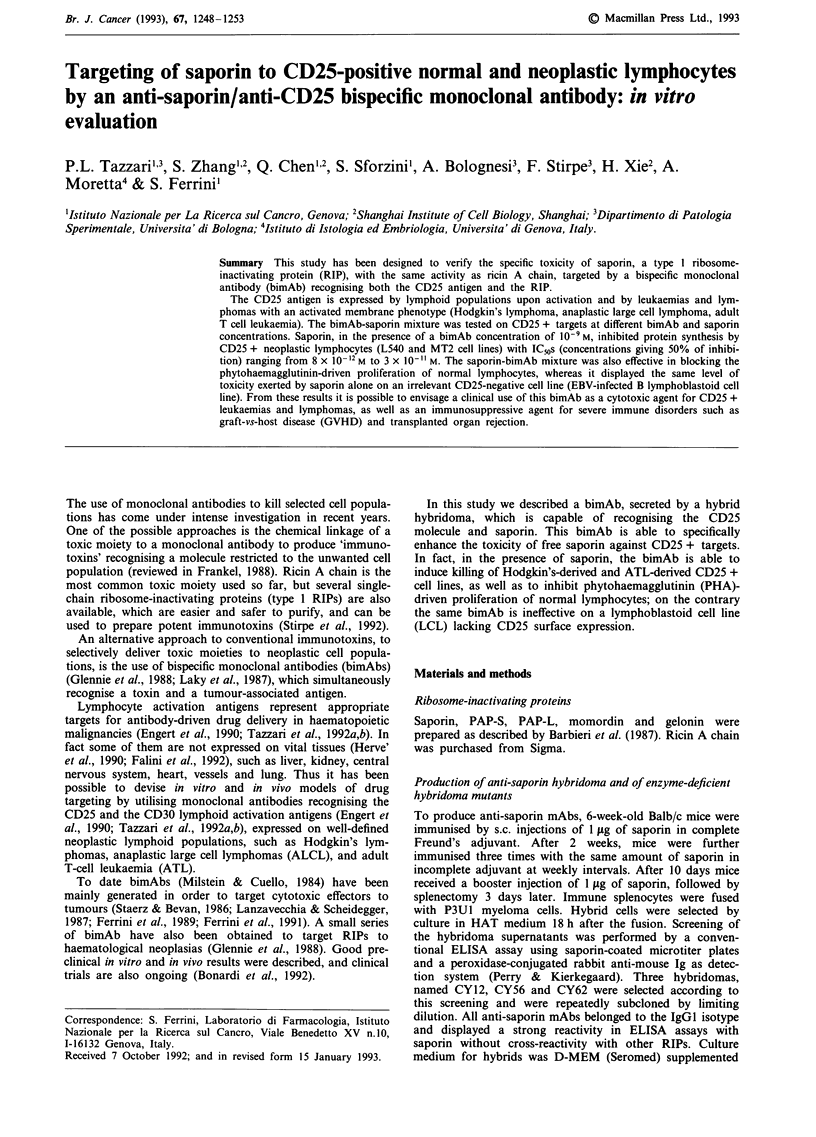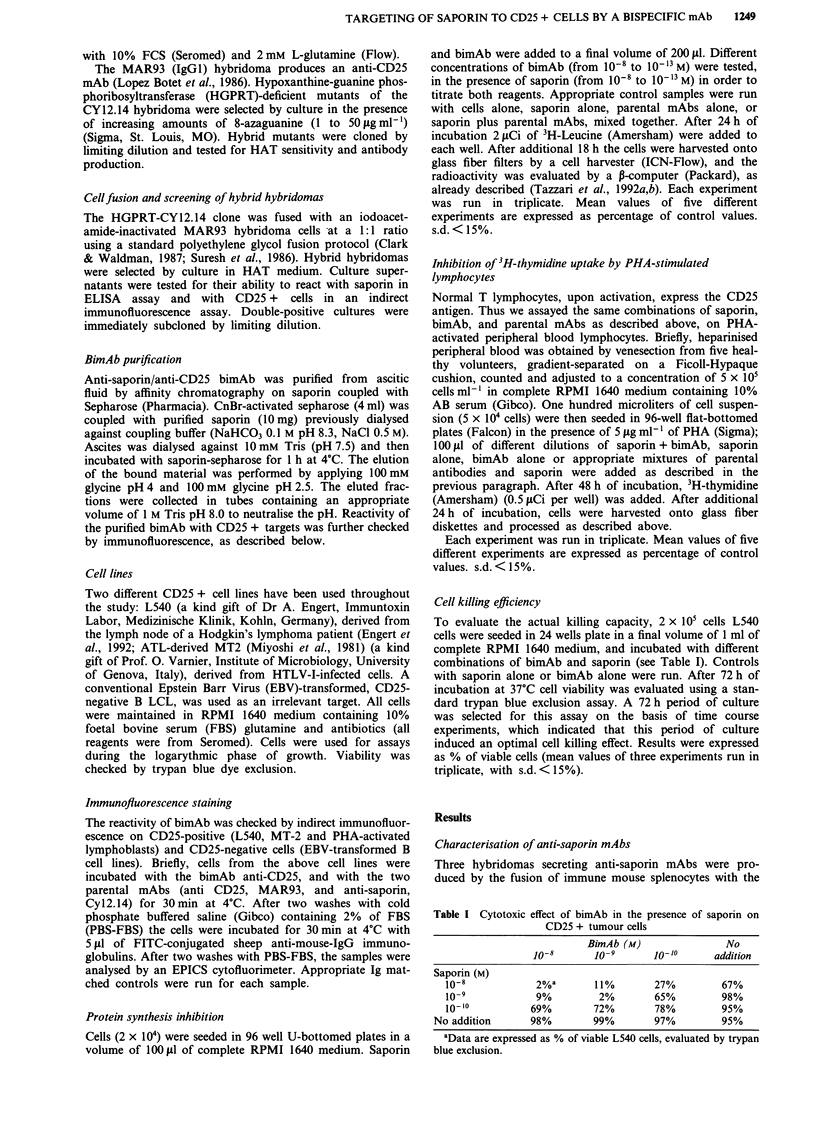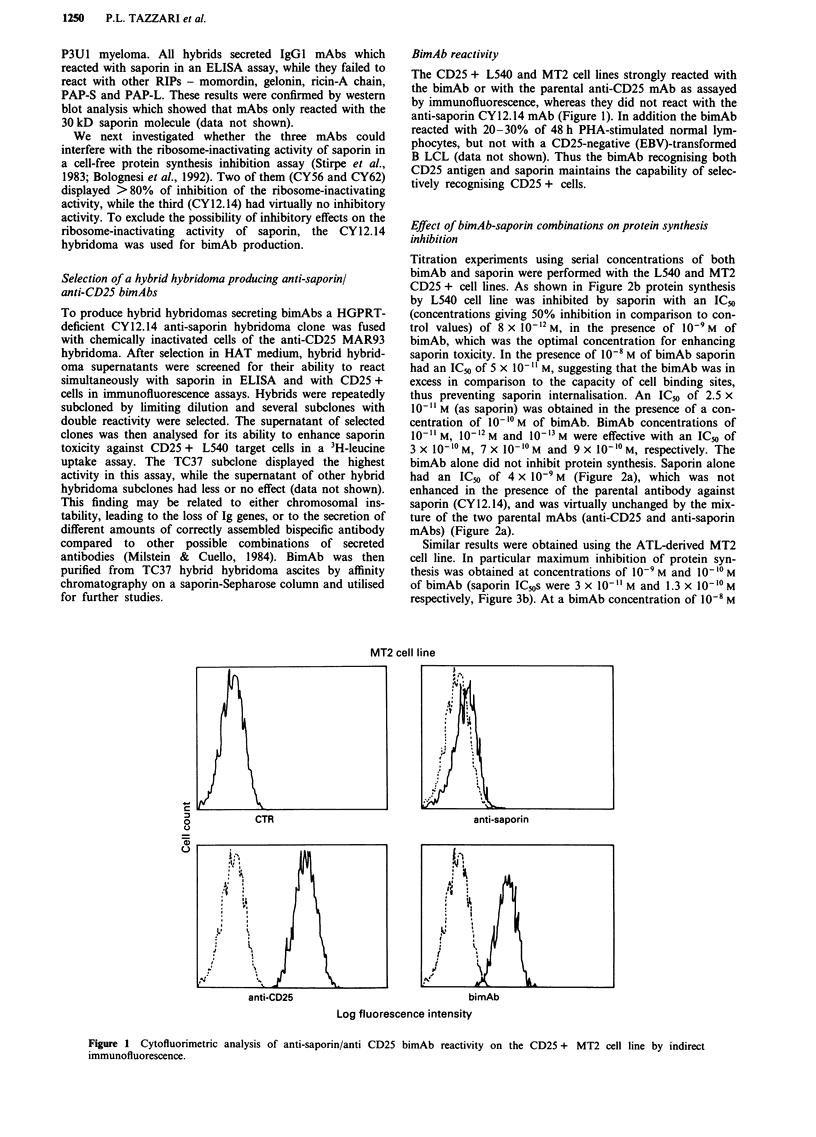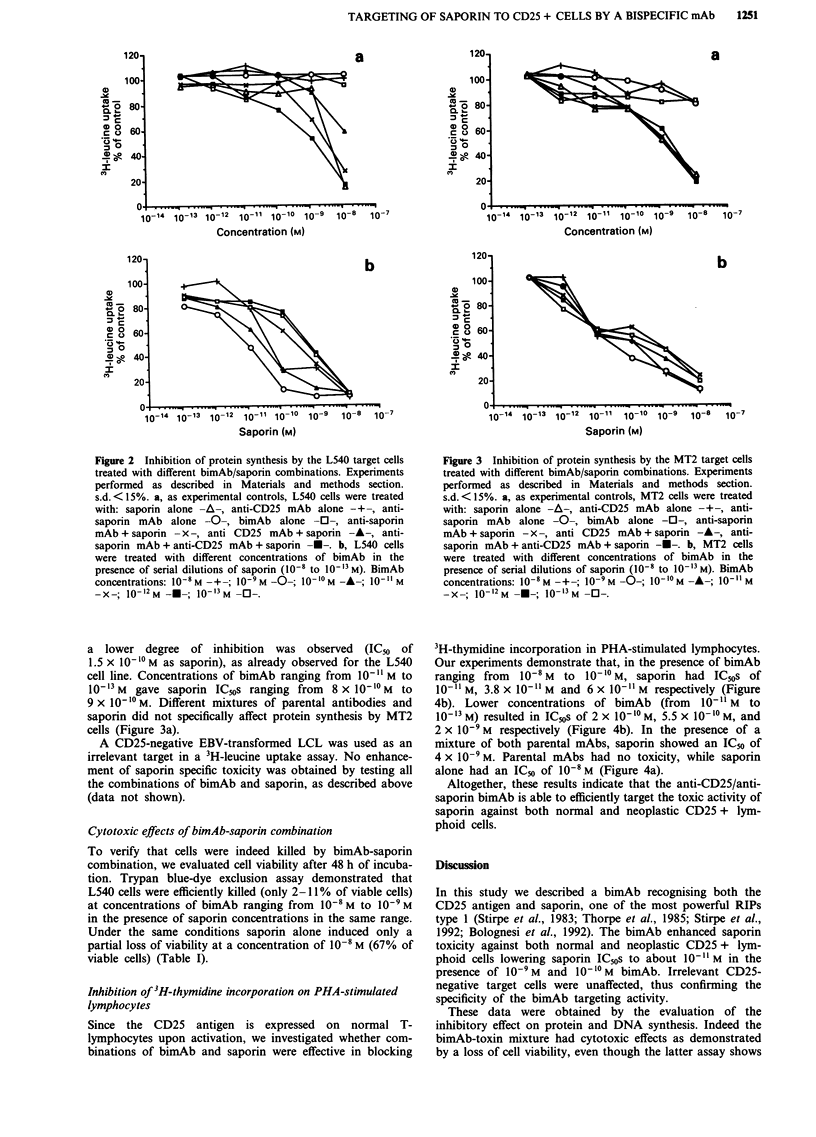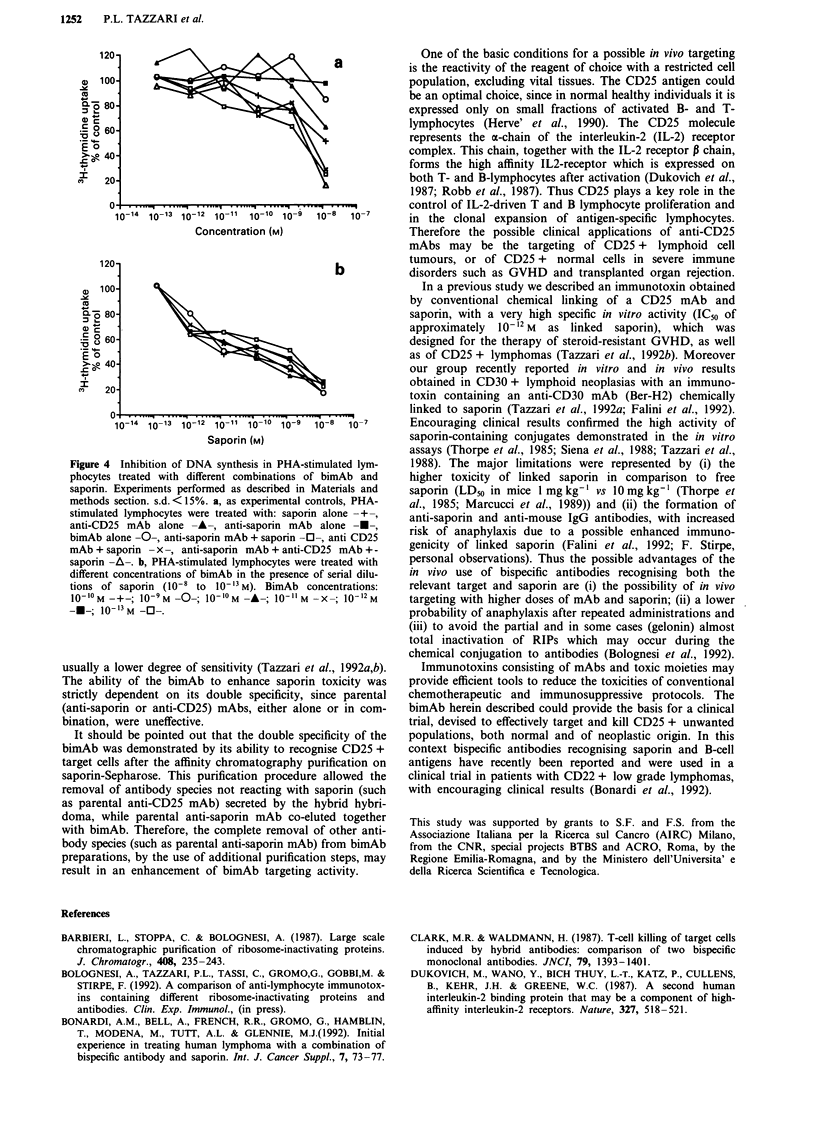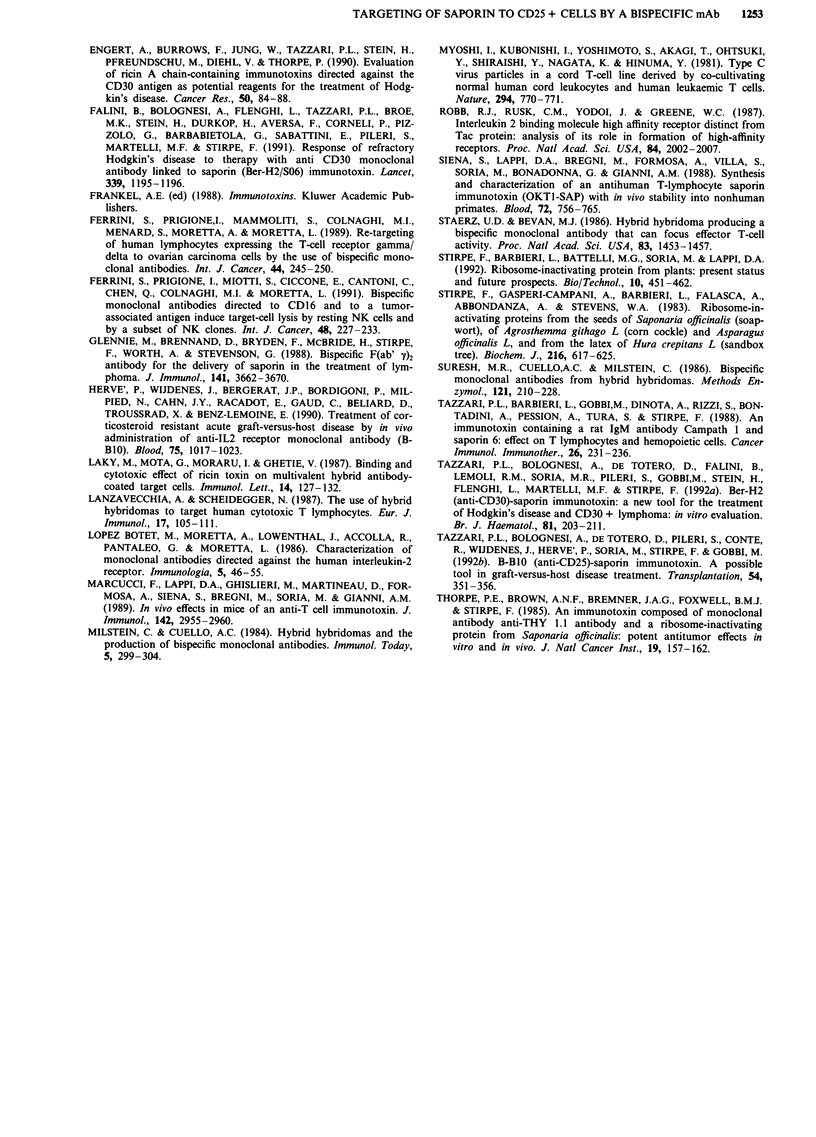# Targeting of saporin to CD25-positive normal and neoplastic lymphocytes by an anti-saporin/anti-CD25 bispecific monoclonal antibody: in vitro evaluation.

**DOI:** 10.1038/bjc.1993.233

**Published:** 1993-06

**Authors:** P. L. Tazzari, S. Zhang, Q. Chen, S. Sforzini, A. Bolognesi, F. Stirpe, H. Xie, A. Moretta, S. Ferrini

**Affiliations:** Istituto Nazionale per La Ricerca sul Cancro, Genova, Italy.

## Abstract

This study has been designed to verify the specific toxicity of saporin, a type 1 ribosome-inactivating protein (RIP), with the same activity as ricin A chain, targeted by a bispecific monoclonal antibody (bimAb) recognising both the CD25 antigen and the RIP. The CD25 antigen is expressed by lymphoid populations upon activation and by leukaemias and lymphomas with an activated membrane phenotype (Hodgkin's lymphoma, anaplastic large cell lymphoma, adult T cell leukaemia). The bimAb-saporin mixture was tested on CD25+ targets at different bimAb and saporin concentrations. Saporin, in the presence of a bimAb concentration of 10(-9) M, inhibited protein synthesis by CD25+ neoplastic lymphocytes (L540 and MT2 cell lines) with IC50S (concentrations giving 50% of inhibition) ranging from 8 x 10(-12) M to 3 x 10(-11) M. The saporin-bimAb mixture was also effective in blocking the phytohaemagglutinin-driven proliferation of normal lymphocytes, whereas it displayed the same level of toxicity exerted by saporin alone on an irrelevant CD25-negative cell line (EBV-infected B lymphoblastoid cell line). From these results it is possible to envisage a clinical use of this bimAb as a cytotoxic agent for CD25+ leukaemias and lymphomas, as well as an immunosuppressive agent for severe immune disorders such as graft-vs-host disease (GVHD) and transplanted organ rejection.


Br. J. Cancer (1993), 67, 1248–1253 © Macmillan Press Ltd., 1993

### Targeting of saporin to CD25-positive normal and neoplastic lymphocytes by an anti-saporin/anti-CD25 bispecific monoclonal antibody: *in vitro* evaluation

P.L. Tazzari[1,3], S. Zhang[1,2], Q. Chen[1,2], S. Sforzini[1], A. Bolognesi[3], F. Stirpe[3], H. Xie[2], A. Moretta[4] & S. Ferrini[1]

[1]*Istituto Nazionale per La Ricerca sul Cancro, Genova;* [2]*Shanghai Institute of Cell Biology, Shanghai;* [3]*Dipartimento di Patologia Sperimentale, Universita' di Bologna;* [4]*Istituto di Istologia ed Embriologia, Universita' di Genova, Italy.*

**Summary** This study has been designed to verify the specific toxicity of saporin, a type 1 ribosome-inactivating protein (RIP), with the same activity as ricin A chain, targeted by a bispecific monoclonal antibody (bimAb) recognising both the CD25 antigen and the RIP.

The CD25 antigen is expressed by lymphoid populations upon activation and by leukaemias and lymphomas with an activated membrane phenotype (Hodgkin's lymphoma, anaplastic large cell lymphoma, adult T cell leukaemia). The bimAb-saporin mixture was tested on CD25 + targets at different bimAb and saporin concentrations. Saporin, in the presence of a bimAb concentration of $10^{-9}$ M, inhibited protein synthesis by CD25 + neoplastic lymphocytes (L540 and MT2 cell lines) with $IC_{50}$s (concentrations giving 50% of inhibition) ranging from $8 \times 10^{-12}$ M to $3 \times 10^{-11}$ M. The saporin-bimAb mixture was also effective in blocking the phytohaemagglutinin-driven proliferation of normal lymphocytes, whereas it displayed the same level of toxicity exerted by saporin alone on an irrelevant CD25-negative cell line (EBV-infected B lymphoblastoid cell line). From these results it is possible to envisage a clinical use of this bimAb as a cytotoxic agent for CD25 + leukaemias and lymphomas, as well as an immunosuppressive agent for severe immune disorders such as graft-*vs*-host disease (GVHD) and transplanted organ rejection.

The use of monoclonal antibodies to kill selected cell populations has come under intense investigation in recent years. One of the possible approaches is the chemical linkage of a toxic moiety to a monoclonal antibody to produce 'immunotoxins' recognising a molecule restricted to the unwanted cell population (reviewed in Frankel, 1988). Ricin A chain is the most common toxic moiety used so far, but several single-chain ribosome-inactivating proteins (type 1 RIPs) are also available, which are easier and safer to purify, and can be used to prepare potent immunotoxins (Stirpe *et al.*, 1992).

An alternative approach to conventional immunotoxins, to selectively deliver toxic moieties to neoplastic cell populations, is the use of bispecific monoclonal antibodies (bimAbs) (Glennie *et al.*, 1988; Laky *et al.*, 1987), which simultaneously recognise a toxin and a tumour-associated antigen.

Lymphocyte activation antigens represent appropriate targets for antibody-driven drug delivery in haematopoietic malignancies (Engert *et al.*, 1990; Tazzari *et al.*, 1992*a*,*b*). In fact some of them are not expressed on vital tissues (Herve' *et al.*, 1990; Falini *et al.*, 1992), such as liver, kidney, central nervous system, heart, vessels and lung. Thus it has been possible to devise *in vitro* and *in vivo* models of drug targeting by utilising monoclonal antibodies recognising the CD25 and the CD30 lymphoid activation antigens (Engert *et al.*, 1990; Tazzari *et al.*, 1992*a*,*b*), expressed on well-defined neoplastic lymphoid populations, such as Hodgkin's lymphomas, anaplastic large cell lymphomas (ALCL), and adult T-cell leukaemia (ATL).

To date bimAbs (Milstein & Cuello, 1984) have been mainly generated in order to target cytotoxic effectors to tumours (Staerz & Bevan, 1986; Lanzavecchia & Scheidegger, 1987; Ferrini *et al.*, 1989; Ferrini *et al.*, 1991). A small series of bimAb have also been obtained to target RIPs to haematological neoplasias (Glennie *et al.*, 1988). Good preclinical *in vitro* and *in vivo* results were described, and clinical trials are also ongoing (Bonardi *et al.*, 1992).

Correspondence: S. Ferrini, Laboratorio di Farmacologia, Istituto Nazionale per la Ricerca sul Cancro, Viale Benedetto XV n.10, I-16132 Genova, Italy.
Received 7 October 1992; and in revised form 15 January 1993.

In this study we described a bimAb, secreted by a hybrid hybridoma, which is capable of recognising the CD25 molecule and saporin. This bimAb is able to specifically enhance the toxicity of free saporin against CD25 + targets. In fact, in the presence of saporin, the bimAb is able to induce killing of Hodgkin's-derived and ATL-derived CD25 + cell lines, as well as to inhibit phytohaemagglutinin (PHA)-driven proliferation of normal lymphocytes; on the contrary the same bimAb is ineffective on a lymphoblastoid cell line (LCL) lacking CD25 surface expression.

### Materials and methods

### *Ribosome-inactivating proteins*

Saporin, PAP-S, PAP-L, momordin and gelonin were prepared as described by Barbieri *et al.* (1987). Ricin A chain was purchased from Sigma.

### *Production of anti-saporin hybridoma and of enzyme-deficient hybridoma mutants*

To produce anti-saporin mAbs, 6-week-old Balb/c mice were immunised by s.c. injections of 1 μg of saporin in complete Freund's adjuvant. After 2 weeks, mice were further immunised three times with the same amount of saporin in incomplete adjuvant at weekly intervals. After 10 days mice received a booster injection of 1 μg of saporin, followed by splenectomy 3 days later. Immune splenocytes were fused with P3U1 myeloma cells. Hybrid cells were selected by culture in HAT medium 18 h after the fusion. Screening of the hybridoma supernatants was performed by a conventional ELISA assay using saporin-coated microtiter plates and a peroxidase-conjugated rabbit anti-mouse Ig as detection system (Perry & Kierkegaard). Three hybridomas, named CY12, CY56 and CY62 were selected according to this screening and were repeatedly subcloned by limiting dilution. All anti-saporin mAbs belonged to the IgG1 isotype and displayed a strong reactivity in ELISA assays with saporin without cross-reactivity with other RIPs. Culture medium for hybrids was D-MEM (Seromed) supplemented with 10% FCS (Seromed) and 2 mM L-glutamine (Flow).

The MAR93 (IgG1) hybridoma produces an anti-CD25 mAb (Lopez Botet *et al.*, 1986). Hypoxanthine-guanine phosphoribosyltransferase (HGPRT)-deficient mutants of the CY12.14 hybridoma were selected by culture in the presence of increasing amounts of 8-azaguanine (1 to 50 $\mu$g ml$^{-1}$) (Sigma, St. Louis, MO). Hybrid mutants were cloned by limiting dilution and tested for HAT sensitivity and antibody production.

*Cell fusion and screening of hybrid hybridomas*

The HGPRT-CY12.14 clone was fused with an iodoacetamide-inactivated MAR93 hybridoma cells at a 1:1 ratio using a standard polyethylene glycol fusion protocol (Clark & Waldman, 1987; Suresh *et al.*, 1986). Hybrid hybridomas were selected by culture in HAT medium. Culture supernatants were tested for their ability to react with saporin in ELISA assay and with CD25+ cells in an indirect immunofluorescence assay. Double-positive cultures were immediately subcloned by limiting dilution.

*BimAb purification*

Anti-saporin/anti-CD25 bimAb was purified from ascitic fluid by affinity chromatography on saporin coupled with Sepharose (Pharmacia). CnBr-activated sepharose (4 ml) was coupled with purified saporin (10 mg) previously dialysed against coupling buffer ($NaHCO_3$ 0.1 M pH 8.3, NaCl 0.5 M). Ascites was dialysed against 10 mM Tris (pH 7.5) and then incubated with saporin-sepharose for 1 h at 4°C. The elution of the bound material was performed by applying 100 mM glycine pH 4 and 100 mM glycine pH 2.5. The eluted fractions were collected in tubes containing an appropriate volume of 1 M Tris pH 8.0 to neutralise the pH. Reactivity of the purified bimAb with CD25 + targets was further checked by immunofluorescence, as described below.

*Cell lines*

Two different CD25 + cell lines have been used throughout the study: L540 (a kind gift of Dr A. Engert, Immuntoxin Labor, Medizinische Klinik, Kohln, Germany), derived from the lymph node of a Hodgkin's lymphoma patient (Engert *et al.*, 1992; ATL-derived MT2 (Miyoshi *et al.*, 1981) (a kind gift of Prof. O. Varnier, Institute of Microbiology, University of Genova, Italy), derived from HTLV-I-infected cells. A conventional Epstein Barr Virus (EBV)-transformed, CD25-negative B LCL, was used as an irrelevant target. All cells were maintained in RPMI 1640 medium containing 10% foetal bovine serum (FBS) glutamine and antibiotics (all reagents were from Seromed). Cells were used for assays during the logarythmic phase of growth. Viability was checked by trypan blue dye exclusion.

*Immunofluorescence staining*

The reactivity of bimAb was checked by indirect immunofluorescence on CD25-positive (L540, MT-2 and PHA-activated lymphoblasts) and CD25-negative cells (EBV-transformed B cell lines). Briefly, cells from the above cell lines were incubated with the bimAb anti-CD25, and with the two parental mAbs (anti CD25, MAR93, and anti-saporin, Cy12.14) for 30 min at 4°C. After two washes with cold phosphate buffered saline (Gibco) containing 2% of FBS (PBS-FBS) the cells were incubated for 30 min at 4°C with 5 $\mu$l of FITC-conjugated sheep anti-mouse-IgG immunoglobulins. After two washes with PBS-FBS, the samples were analysed by an EPICS cytofluorimeter. Appropriate Ig matched controls were run for each sample.

*Protein synthesis inhibition*

Cells ($2 \times 10^4$) were seeded in 96 well U-bottomed plates in a volume of 100 $\mu$l of complete RPMI 1640 medium. Saporin and bimAb were added to a final volume of 200 $\mu$l. Different concentrations of bimAb (from $10^{-8}$ to $10^{-13}$ M) were tested, in the presence of saporin (from $10^{-8}$ to $10^{-13}$ M) in order to titrate both reagents. Appropriate control samples were run with cells alone, saporin alone, parental mAbs alone, or saporin plus parental mAbs, mixed together. After 24 h of incubation 2 $\mu$Ci of $^3$H-Leucine (Amersham) were added to each well. After additional 18 h the cells were harvested onto glass fiber filters by a cell harvester (ICN-Flow), and the radioactivity was evaluated by a $\beta$-computer (Packard), as already described (Tazzari *et al.*, 1992*a,b*). Each experiment was run in triplicate. Mean values of five different experiments are expressed as percentage of control values. s.d. < 15%.

*Inhibition of $^3$H-thymidine uptake by PHA-stimulated lymphocytes*

Normal T lymphocytes, upon activation, express the CD25 antigen. Thus we assayed the same combinations of saporin, bimAb, and parental mAbs as described above, on PHA-activated peripheral blood lymphocytes. Briefly, heparinised peripheral blood was obtained by venesection from five healthy volunteers, gradient-separated on a Ficoll-Hypaque cushion, counted and adjusted to a concentration of $5 \times 10^5$ cells ml$^{-1}$ in complete RPMI 1640 medium containing 10% AB serum (Gibco). One hundred microliters of cell suspension ($5 \times 10^4$ cells) were then seeded in 96-well flat-bottomed plates (Falcon) in the presence of 5 $\mu$g ml$^{-1}$ of PHA (Sigma); 100 $\mu$l of different dilutions of saporin + bimAb, saporin alone, bimAb alone or appropriate mixtures of parental antibodies and saporin were added as described in the previous paragraph. After 48 h of incubation, $^3$H-thymidine (Amersham) (0.5 $\mu$Ci per well) was added. After additional 24 h of incubation, cells were harvested onto glass fiber diskettes and processed as described above.

Each experiment was run in triplicate. Mean values of five different experiments are expressed as percentage of control values. s.d. < 15%.

*Cell killing efficiency*

To evaluate the actual killing capacity, $2 \times 10^5$ cells L540 cells were seeded in 24 wells plate in a final volume of 1 ml of complete RPMI 1640 medium, and incubated with different combinations of bimAb and saporin (see Table I). Controls with saporin alone or bimAb alone were run. After 72 h of incubation at 37°C cell viability was evaluated using a standard trypan blue exclusion assay. A 72 h period of culture was selected for this assay on the basis of time course experiments, which indicated that this period of culture induced an optimal cell killing effect. Results were expressed as % of viable cells (mean values of three experiments run in triplicate, with s.d. < 15%).

### Results

*Characterisation of anti-saporin mAbs*

Three hybridomas secreting anti-saporin mAbs were produced by the fusion of immune mouse splenocytes with the

**Table I** Cytotoxic effect of bimAb in the presence of saporin on CD25 + tumour cells

| | *BimAb (M)* $10^{-8}$ | $10^{-9}$ | $10^{-10}$ | *No addition* |
|---|---|---|---|---|
| Saporin (M) | | | | |
| $10^{-8}$ | 2%[a] | 11% | 27% | 67% |
| $10^{-9}$ | 9% | 2% | 65% | 98% |
| $10^{-10}$ | 69% | 72% | 78% | 95% |
| No addition | 98% | 99% | 97% | 95% |

[a]Data are expressed as % of viable L540 cells, evaluated by trypan blue exclusion.

P3U1 myeloma. All hybrids secreted IgG1 mAbs which reacted with saporin in an ELISA assay, while they failed to react with other RIPs – momordin, gelonin, ricin-A chain, PAP-S and PAP-L. These results were confirmed by western blot analysis which showed that mAbs only reacted with the 30 kD saporin molecule (data not shown).

We next investigated whether the three mAbs could interfere with the ribosome-inactivating activity of saporin in a cell-free protein synthesis inhibition assay (Stirpe *et al.*, 1983; Bolognesi *et al.*, 1992). Two of them (CY56 and CY62) displayed $>80\%$ of inhibition of the ribosome-inactivating activity, while the third (CY12.14) had virtually no inhibitory activity. To exclude the possibility of inhibitory effects on the ribosome-inactivating activity of saporin, the CY12.14 hybridoma was used for bimAb production.

*Selection of a hybrid hybridoma producing anti-saporin/anti-CD25 bimAbs*

To produce hybrid hybridomas secreting bimAbs a HGPRT-deficient CY12.14 anti-saporin hybridoma clone was fused with chemically inactivated cells of the anti-CD25 MAR93 hybridoma. After selection in HAT medium, hybrid hybridoma supernatants were screened for their ability to react simultaneously with saporin in ELISA and with CD25+ cells in immunofluorescence assays. Hybrids were repeatedly subcloned by limiting dilution and several subclones with double reactivity were selected. The supernatant of selected clones was then analysed for its ability to enhance saporin toxicity against CD25+ L540 target cells in a $^{3}$H-leucine uptake assay. The TC37 subclone displayed the highest activity in this assay, while the supernatant of other hybrid hybridoma subclones had less or no effect (data not shown). This finding may be related to either chromosomal instability, leading to the loss of Ig genes, or to the secretion of different amounts of correctly assembled bispecific antibody compared to other possible combinations of secreted antibodies (Milstein & Cuello, 1984). BimAb was then purified from TC37 hybrid hybridoma ascites by affinity chromatography on a saporin-Sepharose column and utilised for further studies.

*BimAb reactivity*

The CD25+ L540 and MT2 cell lines strongly reacted with the bimAb or with the parental anti-CD25 mAb as assayed by immunofluorescence, whereas they did not react with the anti-saporin CY12.14 mAb (Figure 1). In addition the bimAb reacted with 20–30% of 48 h PHA-stimulated normal lymphocytes, but not with a CD25-negative (EBV)-transformed B LCL (data not shown). Thus the bimAb recognising both CD25 antigen and saporin maintains the capability of selectively recognising CD25+ cells.

*Effect of bimAb-saporin combinations on protein synthesis inhibition*

Titration experiments using serial concentrations of both bimAb and saporin were performed with the L540 and MT2 CD25+ cell lines. As shown in Figure 2b protein synthesis by L540 cell line was inhibited by saporin with an $IC_{50}$ (concentrations giving 50% inhibition in comparison to control values) of $8 \times 10^{-12}$ M, in the presence of $10^{-9}$ M of bimAb, which was the optimal concentration for enhancing saporin toxicity. In the presence of $10^{-8}$ M of bimAb saporin had an $IC_{50}$ of $5 \times 10^{-11}$ M, suggesting that the bimAb was in excess in comparison to the capacity of cell binding sites, thus preventing saporin internalisation. An $IC_{50}$ of $2.5 \times 10^{-11}$ M (as saporin) was obtained in the presence of a concentration of $10^{-10}$ M of bimAb. BimAb concentrations of $10^{-11}$ M, $10^{-12}$ M and $10^{-13}$ M were effective with an $IC_{50}$ of $3 \times 10^{-10}$ M, $7 \times 10^{-10}$ M and $9 \times 10^{-10}$ M, respectively. The bimAb alone did not inhibit protein synthesis. Saporin alone had an $IC_{50}$ of $4 \times 10^{-9}$ M (Figure 2a), which was not enhanced in the presence of the parental antibody against saporin (CY12.14), and was virtually unchanged by the mixture of the two parental mAbs (anti-CD25 and anti-saporin mAbs) (Figure 2a).

Similar results were obtained using the ATL-derived MT2 cell line. In particular maximum inhibition of protein synthesis was obtained at concentrations of $10^{-9}$ M and $10^{-10}$ M of bimAb (saporin $IC_{50}$s were $3 \times 10^{-11}$ M and $1.3 \times 10^{-10}$ M respectively, Figure 3b). At a bimAb concentration of $10^{-8}$ M

**Figure 1** Cytofluorimetric analysis of anti-saporin/anti CD25 bimAb reactivity on the CD25+ MT2 cell line by indirect immunofluorescence.

**Figure 2** Inhibition of protein synthesis by the L540 target cells treated with different bimAb/saporin combinations. Experiments performed as described in Materials and methods section. s.d. < 15%. **a**, as experimental controls, L540 cells were treated with: saporin alone -Δ-, anti-CD25 mAb alone -+-, anti-saporin mAb alone -O-, bimAb alone -□-, anti-saporin mAb + saporin -×-, anti CD25 mAb + saporin -▲-, anti-saporin mAb + anti-CD25 mAb + saporin -■-. **b**, L540 cells were treated with different concentrations of bimAb in the presence of serial dilutions of saporin ($10^{-8}$ to $10^{-13}$ M). BimAb concentrations: $10^{-8}$ M -+-; $10^{-9}$ M -O-; $10^{-10}$ M -▲-; $10^{-11}$ M -×-; $10^{-12}$ M -■-; $10^{-13}$ M -□-.

**Figure 3** Inhibition of protein synthesis by the MT2 target cells treated with different bimAb/saporin combinations. Experiments performed as described in Materials and methods section. s.d. < 15%. **a**, as experimental controls, MT2 cells were treated with: saporin alone -Δ-, anti-CD25 mAb alone -+-, anti-saporin mAb alone -O-, bimAb alone -□-, anti-saporin mAb + saporin -×-, anti CD25 mAb + saporin -▲-, anti-saporin mAb + anti-CD25 mAb + saporin -■-. **b**, MT2 cells were treated with different concentrations of bimAb in the presence of serial dilutions of saporin ($10^{-8}$ to $10^{-13}$ M). BimAb concentrations: $10^{-8}$ M -+-; $10^{-9}$ M -O-; $10^{-10}$ M -▲-; $10^{-11}$ M -×-; $10^{-12}$ M -■-; $10^{-13}$ M -□-.

a lower degree of inhibition was observed ($IC_{50}$ of $1.5 \times 10^{-10}$ M as saporin), as already observed for the L540 cell line. Concentrations of bimAb ranging from $10^{-11}$ M to $10^{-13}$ M gave saporin $IC_{50}$s ranging from $8 \times 10^{-10}$ M to $9 \times 10^{-10}$ M. Different mixtures of parental antibodies and saporin did not specifically affect protein synthesis by MT2 cells (Figure 3a).

A CD25-negative EBV-transformed LCL was used as an irrelevant target in a $^3$H-leucine uptake assay. No enhancement of saporin specific toxicity was obtained by testing all the combinations of bimAb and saporin, as described above (data not shown).

### *Cytotoxic effects of bimAb-saporin combination*

To verify that cells were indeed killed by bimAb-saporin combination, we evaluated cell viability after 48 h of incubation. Trypan blue-dye exclusion assay demonstrated that L540 cells were efficiently killed (only 2–11% of viable cells) at concentrations of bimAb ranging from $10^{-8}$ M to $10^{-9}$ M in the presence of saporin concentrations in the same range. Under the same conditions saporin alone induced only a partial loss of viability at a concentration of $10^{-8}$ M (67% of viable cells) (Table I).

### *Inhibition of $^3$H-thymidine incorporation on PHA-stimulated lymphocytes*

Since the CD25 antigen is expressed on normal T-lymphocytes upon activation, we investigated whether combinations of bimAb and saporin were effective in blocking $^3$H-thymidine incorporation in PHA-stimulated lymphocytes. Our experiments demonstrate that, in the presence of bimAb ranging from $10^{-8}$ M to $10^{-10}$ M, saporin had $IC_{50}$s of $10^{-11}$ M, $3.8 \times 10^{-11}$ M and $6 \times 10^{-11}$ M respectively (Figure 4b). Lower concentrations of bimAb (from $10^{-11}$ M to $10^{-13}$ M) resulted in $IC_{50}$s of $2 \times 10^{-10}$ M, $5.5 \times 10^{-10}$ M, and $2 \times 10^{-9}$ M respectively (Figure 4b). In the presence of a mixture of both parental mAbs, saporin showed an $IC_{50}$ of $4 \times 10^{-9}$ M. Parental mAbs had no toxicity, while saporin alone had an $IC_{50}$ of $10^{-8}$ M (Figure 4a).

Altogether, these results indicate that the anti-CD25/anti-saporin bimAb is able to efficiently target the toxic activity of saporin against both normal and neoplastic CD25 + lymphoid cells.

### Discussion

In this study we described a bimAb recognising both the CD25 antigen and saporin, one of the most powerful RIPs type 1 (Stirpe *et al.*, 1983; Thorpe *et al.*, 1985; Stirpe *et al.*, 1992; Bolognesi *et al.*, 1992). The bimAb enhanced saporin toxicity against both normal and neoplastic CD25 + lymphoid cells lowering saporin $IC_{50}$s to about $10^{-11}$ M in the presence of $10^{-9}$ M and $10^{-10}$ M bimAb. Irrelevant CD25-negative target cells were unaffected, thus confirming the specificity of the bimAb targeting activity.

These data were obtained by the evaluation of the inhibitory effect on protein and DNA synthesis. Indeed the bimAb-toxin mixture had cytotoxic effects as demonstrated by a loss of cell viability, even though the latter assay shows

**Figure 4** Inhibition of DNA synthesis in PHA-stimulated lymphocytes treated with different combinations of bimAb and saporin. Experiments performed as described in Materials and methods section. s.d. < 15%. **a**, as experimental controls, PHA-stimulated lymphocytes were treated with: saporin alone -+-, anti-CD25 mAb alone -▲-, anti-saporin mAb alone -■-, bimAb alone -O-, anti-saporin mAb + saporin -□-, anti CD25 mAb + saporin -×-, anti-saporin mAb + anti-CD25 mAb + saporin -△-. **b**, PHA-stimulated lymphocytes were treated with different concentrations of bimAb in the presence of serial dilutions of saporin ($10^{-8}$ to $10^{-13}$ M). BimAb concentrations: $10^{-10}$ M -+-; $10^{-9}$ M -O-; $10^{-10}$ M -▲-; $10^{-11}$ M -×-; $10^{-12}$ M -■-; $10^{-13}$ M -□-.

usually a lower degree of sensitivity (Tazzari *et al.*, 1992*a*,*b*). The ability of the bimAb to enhance saporin toxicity was strictly dependent on its double specificity, since parental (anti-saporin or anti-CD25) mAbs, either alone or in combination, were uneffective.

It should be pointed out that the double specificity of the bimAb was demonstrated by its ability to recognise CD25 + target cells after the affinity chromatography purification on saporin-Sepharose. This purification procedure allowed the removal of antibody species not reacting with saporin (such as parental anti-CD25 mAb) secreted by the hybrid hybridoma, while parental anti-saporin mAb co-eluted together with bimAb. Therefore, the complete removal of other antibody species (such as parental anti-saporin mAb) from bimAb preparations, by the use of additional purification steps, may result in an enhancement of bimAb targeting activity.

One of the basic conditions for a possible *in vivo* targeting is the reactivity of the reagent of choice with a restricted cell population, excluding vital tissues. The CD25 antigen could be an optimal choice, since in normal healthy individuals it is expressed only on small fractions of activated B- and T-lymphocytes (Herve' *et al.*, 1990). The CD25 molecule represents the α-chain of the interleukin-2 (IL-2) receptor complex. This chain, together with the IL-2 receptor β chain, forms the high affinity IL2-receptor which is expressed on both T- and B-lymphocytes after activation (Dukovich *et al.*, 1987; Robb *et al.*, 1987). Thus CD25 plays a key role in the control of IL-2-driven T and B lymphocyte proliferation and in the clonal expansion of antigen-specific lymphocytes. Therefore the possible clinical applications of anti-CD25 mAbs may be the targeting of CD25 + lymphoid cell tumours, or of CD25 + normal cells in severe immune disorders such as GVHD and transplanted organ rejection.

In a previous study we described an immunotoxin obtained by conventional chemical linking of a CD25 mAb and saporin, with a very high specific *in vitro* activity ($IC_{50}$ of approximately $10^{-12}$ M as linked saporin), which was designed for the therapy of steroid-resistant GVHD, as well as of CD25 + lymphomas (Tazzari *et al.*, 1992*b*). Moreover our group recently reported *in vitro* and *in vivo* results obtained in CD30 + lymphoid neoplasias with an immunotoxin containing an anti-CD30 mAb (Ber-H2) chemically linked to saporin (Tazzari *et al.*, 1992*a*; Falini *et al.*, 1992). Encouraging clinical results confirmed the high activity of saporin-containing conjugates demonstrated in the *in vitro* assays (Thorpe *et al.*, 1985; Siena *et al.*, 1988; Tazzari *et al.*, 1988). The major limitations were represented by (i) the higher toxicity of linked saporin in comparison to free saporin ($LD_{50}$ in mice 1 mg kg$^{-1}$ *vs* 10 mg kg$^{-1}$ (Thorpe *et al.*, 1985; Marcucci *et al.*, 1989)) and (ii) the formation of anti-saporin and anti-mouse IgG antibodies, with increased risk of anaphylaxis due to a possible enhanced immunogenicity of linked saporin (Falini *et al.*, 1992; F. Stirpe, personal observations). Thus the possible advantages of the *in vivo* use of bispecific antibodies recognising both the relevant target and saporin are (i) the possibility of *in vivo* targeting with higher doses of mAb and saporin; (ii) a lower probability of anaphylaxis after repeated administrations and (iii) to avoid the partial and in some cases (gelonin) almost total inactivation of RIPs which may occur during the chemical conjugation to antibodies (Bolognesi *et al.*, 1992).

Immunotoxins consisting of mAbs and toxic moieties may provide efficient tools to reduce the toxicities of conventional chemotherapeutic and immunosuppressive protocols. The bimAb herein described could provide the basis for a clinical trial, devised to effectively target and kill CD25 + unwanted populations, both normal and of neoplastic origin. In this context bispecific antibodies recognising saporin and B-cell antigens have recently been reported and were used in a clinical trial in patients with CD22 + low grade lymphomas, with encouraging clinical results (Bonardi *et al.*, 1992).

This study was supported by grants to S.F. and F.S. from the Associazione Italiana per la Ricerca sul Cancro (AIRC) Milano, from the CNR, special projects BTBS and ACRO, Roma, by the Regione Emilia-Romagna, and by the Ministero dell'Universita' e della Ricerca Scientifica e Tecnologica.

### References

BARBIERI, L., STOPPA, C. & BOLOGNESI, A. (1987). Large scale chromatographic purification of ribosome-inactivating proteins. *J. Chromatogr.*, **408**, 235–243.

BOLOGNESI, A., TAZZARI, P.L., TASSI, C., GROMO,G., GOBBI,M. & STIRPE, F. (1992). A comparison of anti-lymphocyte immunotoxins containing different ribosome-inactivating proteins and antibodies. *Clin. Exp. Immunol.*, (in press).

BONARDI, A.M., BELL, A., FRENCH, R.R., GROMO, G., HAMBLIN, T., MODENA, M., TUTT, A.L. & GLENNIE, M.J.(1992). Initial experience in treating human lymphoma with a combination of bispecific antibody and saporin. *Int. J. Cancer Suppl.*, **7**, 73–77.

CLARK, M.R. & WALDMANN, H. (1987). T-cell killing of target cells induced by hybrid antibodies: comparison of two bispecific monoclonal antibodies. *JNCI*, **79**, 1393–1401.

DUKOVICH, M., WANO, Y., BICH THUY, L.-T., KATZ, P., CULLENS, B., KEHR, J.H. & GREENE, W.C. (1987). A second human interleukin-2 binding protein that may be a component of high-affinity interleukin-2 receptors. *Nature*, **327**, 518–521.

ENGERT, A., BURROWS, F., JUNG, W., TAZZARI, P.L., STEIN, H., PFREUNDSCHU, M., DIEHL, V. & THORPE, P. (1990). Evaluation of ricin A chain-containing immunotoxins directed against the CD30 antigen as potential reagents for the treatment of Hodgkin's disease. *Cancer Res.*, **50,** 84–88.

FALINI, B., BOLOGNESI, A., FLENGHI, L., TAZZARI, P.L., BROE, M.K., STEIN, H., DURKOP, H., AVERSA, F., CORNELI, P., PIZZOLO, G., BARBABIETOLA, G., SABATTINI, E., PILERI, S., MARTELLI, M.F. & STIRPE, F. (1991). Response of refractory Hodgkin's disease to therapy with anti CD30 monoclonal antibody linked to saporin (Ber-H2/S06) immunotoxin. *Lancet*, **339,** 1195–1196.

FRANKEL, A.E. (ed) (1988). *Immunotoxins*. Kluwer Academic Publishers.

FERRINI, S., PRIGIONE,I., MAMMOLITI, S., COLNAGHI, M.I., MENARD, S., MORETTA, A. & MORETTA, L. (1989). Re-targeting of human lymphocytes expressing the T-cell receptor gamma/delta to ovarian carcinoma cells by the use of bispecific monoclonal antibodies. *Int. J. Cancer*, **44,** 245–250.

FERRINI, S., PRIGIONE, I., MIOTTI, S., CICCONE, E., CANTONI, C., CHEN, Q., COLNAGHI, M.I. & MORETTA, L. (1991). Bispecific monoclonal antibodies directed to CD16 and to a tumor-associated antigen induce target-cell lysis by resting NK cells and by a subset of NK clones. *Int. J. Cancer*, **48,** 227–233.

GLENNIE, M., BRENNAND, D., BRYDEN, F., MCBRIDE, H., STIRPE, F., WORTH, A. & STEVENSON, G. (1988). Bispecific F(ab' $\gamma$)$_2$ antibody for the delivery of saporin in the treatment of lymphoma. *J. Immunol.*, **141,** 3662–3670.

HERVE', P., WIJDENES, J., BERGERAT, J.P., BORDIGONI, P., MILPIED, N., CAHN, J.Y., RACADOT, E., GAUD, C., BELIARD, D., TROUSSRAD, X. & BENZ-LEMOINE, E. (1990). Treatment of corticosteroid resistant acute graft-versus-host disease by *in vivo* administration of anti-IL2 receptor monoclonal antibody (B-B10). *Blood*, **75,** 1017–1023.

LAKY, M., MOTA, G., MORARU, I. & GHETIE, V. (1987). Binding and cytotoxic effect of ricin toxin on multivalent hybrid antibody-coated target cells. *Immunol. Lett.*, **14,** 127–132.

LANZAVECCHIA, A. & SCHEIDEGGER, N. (1987). The use of hybrid hybridomas to target human cytotoxic T lymphocytes. *Eur. J. Immunol.*, **17,** 105–111.

LOPEZ BOTET, M., MORETTA, A., LOWENTHAL, J., ACCOLLA, R., PANTALEO, G. & MORETTA, L. (1986). Characterization of monoclonal antibodies directed against the human interleukin-2 receptor. *Immunologia*, **5,** 46–55.

MARCUCCI, F., LAPPI, D.A., GHISLIERI, M., MARTINEAU, D., FORMOSA, A., SIENA, S., BREGNI, M., SORIA, M. & GIANNI, A.M. (1989). *In vivo* effects in mice of an anti-T cell immunotoxin. *J. Immunol.*, **142,** 2955–2960.

MILSTEIN, C. & CUELLO, A.C. (1984). Hybrid hybridomas and the production of bispecific monoclonal antibodies. *Immunol. Today*, **5,** 299–304.

MYOSHI, I., KUBONISHI, I., YOSHIMOTO, S., AKAGI, T., OHTSUKI, Y., SHIRAISHI, Y., NAGATA, K. & HINUMA, Y. (1981). Type C virus particles in a cord T-cell line derived by co-cultivating normal human cord leukocytes and human leukaemic T cells. *Nature*, **294,** 770–771.

ROBB, R.J., RUSK, C.M., YODOI, J. & GREENE, W.C. (1987). Interleukin 2 binding molecule high affinity receptor distinct from Tac protein: analysis of its role in formation of high-affinity receptors. *Proc. Natl Acad. Sci. USA*, **84,** 2002–2007.

SIENA, S., LAPPI, D.A., BREGNI, M., FORMOSA, A., VILLA, S., SORIA, M., BONADONNA, G. & GIANNI, A.M. (1988). Synthesis and characterization of an antihuman T-lymphocyte saporin immunotoxin (OKT1-SAP) with *in vivo* stability into nonhuman primates. *Blood*, **72,** 756–765.

STAERZ, U.D. & BEVAN, M.J. (1986). Hybrid hybridoma producing a bispecific monoclonal antibody that can focus effector T-cell activity. *Proc. Natl Acad. Sci. USA*, **83,** 1453–1457.

STIRPE, F., BARBIERI, L., BATTELLI, M.G., SORIA, M. & LAPPI, D.A. (1992). Ribosome-inactivating protein from plants: present status and future prospects. *Bio/Technol.*, **10,** 451–462.

STIRPE, F., GASPERI-CAMPANI, A., BARBIERI, L., FALASCA, A., ABBONDANZA, A. & STEVENS, W.A. (1983). Ribosome-inactivating proteins from the seeds of *Saponaria officinalis* (soapwort), of *Agrosthemma githago L* (corn cockle) and *Asparagus officinalis L*, and from the latex of *Hura crepitans L* (sandbox tree). *Biochem. J.*, **216,** 617–625.

SURESH, M.R., CUELLO,A.C. & MILSTEIN, C. (1986). Bispecific monoclonal antibodies from hybrid hybridomas. *Methods Enzymol.*, **121,** 210–228.

TAZZARI, P.L., BARBIERI, L., GOBBI,M., DINOTA, A., RIZZI, S., BONTADINI, A., PESSION, A., TURA, S. & STIRPE, F. (1988). An immunotoxin containing a rat IgM antibody Campath 1 and saporin 6: effect on T lymphocytes and hemopoietic cells. *Cancer Immunol. Immunother.*, **26,** 231–236.

TAZZARI, P.L., BOLOGNESI, A., DE TOTERO, D., FALINI, B., LEMOLI, R.M., SORIA, M.R., PILERI, S., GOBBI,M., STEIN, H., FLENGHI, L., MARTELLI, M.F. & STIRPE, F. (1992*a*). Ber-H2 (anti-CD30)-saporin immunotoxin: a new tool for the treatment of Hodgkin's disease and CD30 + lymphoma: *in vitro* evaluation. *Br. J. Haematol.*, **81,** 203–211.

TAZZARI, P.L., BOLOGNESI, A., DE TOTERO, D., PILERI, S., CONTE, R., WIJDENES, J., HERVE', P., SORIA, M., STIRPE, F. & GOBBI, M. (1992*b*). B-B10 (anti-CD25)-saporin immunotoxin. A possible tool in graft-versus-host disease treatment. *Transplantation*, **54,** 351–356.

THORPE, P.E., BROWN, A.N.F., BREMNER, J.A.G., FOXWELL, B.M.J. & STIRPE, F. (1985). An immunotoxin composed of monoclonal antibody anti-THY 1.1 antibody and a ribosome-inactivating protein from *Saponaria officinalis*: potent antitumor effects *in vitro* and *in vivo*. *J. Natl Cancer Inst.*, **19,** 157–162.